# Retinal Pigment Epithelium and Photoreceptor Preconditioning Protection Requires Docosanoid Signaling

**DOI:** 10.1007/s10571-017-0565-2

**Published:** 2017-11-24

**Authors:** Eric J. Knott, William C. Gordon, Bokkyoo Jun, Khanh Do, Nicolas G. Bazan

**Affiliations:** Neuroscience Center of Excellence, School of Medicine, Louisiana State University Health New Orleans, New Orleans, LA 70112 USA

**Keywords:** Preconditioning, Docosahexaenoic acid, Docosanoids, Neuroprotectin D1, Retinal pigment epithelial cells, Photoreceptor cells

## Abstract

Omega-3 and omega-6 polyunsaturated fatty acids (PUFAs) are necessary for functional cell integrity. Preconditioning (PC), as we define it, is an acquired protection or resilience by a cell, tissue, or organ to a lethal stimulus enabled by a previous sublethal stressor or stimulus. In this study, we provide evidence that the omega-3 fatty acid docosahexaenoic acid (DHA) and its derivatives, the docosanoids 17-hydroxy docosahexaenoic acid (17-HDHA) and neuroprotectin D1 (NPD1), facilitate cell survival in both in vitro and in vivo models of retinal PC. We also demonstrate that PC requires the enzyme 15-lipoxygenase-1 (15-LOX-1), which synthesizes 17-HDHA and NPD1, and that this is specific to docosanoid signaling despite the concomitant release of the omega-6 arachidonic acid and eicosanoid synthesis. These findings advocate that DHA and docosanoids are protective enablers of PC in photoreceptor and retinal pigment epithelial cells.

**Electronic supplementary material:**

The online version of this article (10.1007/s10571-017-0565-2) contains supplementary material, which is available to authorized users.

## Introduction

Omega-3 polyunsaturated fatty acids (PUFAs) and their enzymatic metabolic derivatives, docosanoids, display potent anti-inflammatory and pro-resolving properties, in contrast to the pro-inflammatory actions of omega-6 PUFA derivatives. Omega-3 and omega-6 PUFAs are distributed differentially to organs and intracellular membranes, and are necessary structural and functional components of cellular membranes (Bazan et al. 2011a). The omega-3 fatty acid family member docosahexaenoic acid (DHA) is avidly retained in the central nervous system (CNS), particularly in photoreceptor cells (PRCs) and synaptic membranes, and it participates in vision, neuroprotection, successful aging, and memory (Anderson et al. 1992). DHA is the precursor of docosanoids, biologically-active mediators that are made ‘on demand’ when factors threaten to disrupt cellular homeostasis (Bazan 2006a, 2007). Under these conditions, the release of DHA takes place via phospholipase A2 (PLA_2_) (Sun et al. 2010). DHA is then further converted metabolically by 15-lipoxygenase-1 (15-LOX-1) to produce the hydroxyl derivatives 17-hydroperoxy-docosahexaenoic acid (17-HpDHA), resolvins, and neuroprotectin D1 (NPD1), all of which regulate neuroprotective, anti-inflammatory, and anti-angiogenic bioactivity (Bazan et al. 1984) (electronic supplementary Fig. 1).

A preconditioning (PC) stimulus is a sublethal or pharmacologic stressor that activates a counter-regulatory protective response to a future lethal stimulus. PC, as we define it, is the response to a PC-stimulus responsible for the protection or resilience by a cell, tissue, or organ to a lethal stimulus. There are several types of PC, which are currently characterized by the stimulus that prompts the protective response (e.g. ischemic, light PC [LPC], hyperthermia, etc.). Mechanistic studies link protective PC effects to trophic factor bioactivity (Ueki et al. 2009), phosphorylation of extracellular signal-regulated kinase (ERK) 1 and 2, modulation of the pre-mitochondrial protein Bcl-xL (Káldi et al. 2003; Li et al. 2003), and activation of protein kinase C (PKC) in photoreceptor and cardiac cells (Dreixler et al. 2008). It also has been reported that the protective actions of PC correlate with elevated non-esterified arachidonic acid (AA), linoleic acid, and lipoxygenase activity during ischemic PC (IPC) of the rat myocardium (Murphy et al. 1995; Starkopf et al. 1998), suggesting that AA lipoxygenase metabolites are involved in IPC via PLA_2_ activation.

DHA has also been reported to be released in response to ischemic myocardium PC (Murphy et al. 1995), and we have shown that DHA is increased in response to, and protects the brain from, ischemia reperfusion (Belayev et al. 2009). It is known that DHA is a substrate of both PLA_2_ and lipoxygenase activity, suggesting that DHA and docosanoids are also involved in the protective phase of PC mechanisms (Bazan et al. 2010). Because there are only a few published articles that evaluate retina or retinal cells and their responses during PC (Li et al. 2001, 2003; Casson et al. 2003; Sharma et al. 2009; Chollangi et al. 2009), we were encouraged to investigate whether DHA and docosanoids are involved in these protective PC-responses.

In this study, we demonstrate that the free pool size of the essential omega-3 fatty acid DHA is increased to form docosanoids, which play a critical role in survival signaling mechanisms during the protective actions of both in vitro and in vivo retinal PC. We show that, in human retinal pigment epithelial (RPE) cells and PRCs, this resilience is mitigated through 15-LOX-1. We also demonstrate that the neurotrophin pigment epithelium-derived factor (PEDF), which also stimulates docosanoid production, is involved. Moreover, we show that NPD1 prevents the loss of protection bestowed by 15-LOX-1 inhibition in vitro and protects PRCs from light damage in vivo, further signifying that DHA and its docosanoid metabolites are pivotal during RPE and photoreceptor PC.

## Materials and Methods

### Animal Experiments

All animal experiments were conducted according to protocols approved by the Animal Research Committee of Louisiana State University (LSU) Health New Orleans and the National Institutes of Health (NIH) Guide for the Care and Use of Laboratory Animals. Male Sprague–Dawley rats (150–175 g) were obtained from Charles River Laboratories (Wilmington, MA, USA) and maintained in the LSU Health New Orleans animal facility on a 12/12 h light/dark cycle (lights were turned on at 0600 h and turned off at 1800 h) at an average illumination of 40 Lux (measured at the level of bedding in the center of the cages, which were maintained in a ventilation rack). Rat gender was selected to coincide with published studies on PC, all of which used only males. Animals were fed normal rat chow and supplied with water ad libitum.

### Retinal Light Preconditioning (LPC)

Male Sprague–Dawley rats (150–175 g) were anesthetized with ketamine and xylazine. For baseline and post-light damage outer retinal thickness, measurements were determined by spectral domain-optical coherence tomography (SD-OCT) and defined as the distance between the photoreceptor synaptic terminals and Bruch’s membrane. Tropicamide ophthalmic solution (1%) was applied to both eyes prior to moderate light exposure (12 h on/12 h off for 3 days at 1200 Lux). Post-moderate light exposure animals were allowed to recover in complete darkness for 24 h prior to lethal bright light exposure. Retinas were also collected and analyzed via liquid chromatography-electrospray-ionization-tandem mass spectrometry (LC-ESI-MS/MS) during day 1 of the moderate to light PC.

### Retinal Ischemic PC (IPC)

Male Sprague–Dawley rats (150–175 g) were anesthetized with ketamine and xylazine and imaged by SD-OCT for baseline, post-light damage, and outer retinal thickness. Corneal analgesia was achieved using 10 μL of 0.5% proparacaine (Allergan, Humacao, Puerto Rico), and body temperature was maintained between 36.5 and 37.0 °C using a heating blanket (Harvard Apparatus, Natick, MA, USA). For PC, intraocular pressure (IOP) was increased to 122 mmHg by inserting a 27-gauge hypodermic needle into the anterior chamber. The increased pressure was maintained for 5 min while the needle was connected to a saline reservoir raised 1.524 m above the animal. Increased IOP (122 mgHg) was determined by hydrostatic calculation, and then confirmed and monitored using a tonometer. In sham-treated, non-conditioned animals, IOP was maintained between 10 and 15 mmHg. The contralateral eye of each animal served as the non-ischemic control.

### Lethal Light Exposure

Male Sprague–Dawley rats (150–175 g) were dark-adapted for 24 h, and tropicamide ophthalmic solution (1%) was applied to both eyes prior to lethal light exposure (white fluorescent light, 18 kLux for 5 h). Some retinas were collected at various time points for lipid LC–ESI-MS/MS analysis, while others were SD-OCT imaged before and after to verify the degree of PRC degeneration.

### Unbiased Cell Survival Quantification by Morphonuclear Analysis Imaging Method (MAIM)

A 4 × 4 tile mosaic with a total area of 3.415 mm^2^ was acquired from the center of each 48-well plate using a Zeiss 510 Meta laser confocal microscope with a 10× objective. Following 10 min of Hoechst 33258 staining, 12-bit images were acquired by 405 nm laser excitation. Images were imported into NIH image analysis software (ImageJ v1.48) and batch-processed using a modified macro as described previously (Stark and Bazan 2011). The cut-off size for the pyknosis was chosen empirically and was based on the nuclear size distributions from the populations of cells subjected to increasing concentrations of hydrogen peroxide (H_2_O_2_) and tumor necrosis factor (TNF)-α. Objects with areas <18 μm^2^ were considered to be fragmented nuclei or extraneous debris. Objects >2.5 times the average naive nuclear size were assumed to be caused by two or more nuclei in contact. We estimated the number of nuclei in overlapping clusters by dividing the area of the cluster by the average nuclear size from a population of naive cells, and the quotient of this operation was tallied with the non-pyknotic nuclei since clustering tended to only occur during naive and non-pyknotic outcomes.

### ARPE-19 and Human Primary Retinal Pigment Epithelial (RPE) Cells

ARPE-19 cells, a human RPE cell line, were obtained from American Type Culture Collection, Manassas, VA, USA. Routine analyses of our RPE cell lines are performed in our laboratory to ensure that they retain their RPE cell characteristics (electronic supplementary Fig. 4). Human primary RPE cells from donor eyes of a 19-year-old Caucasian male without eye pathology were obtained from the National Disease Research Interchange (NDRI) within 24 h after death (head trauma). It is important to note that patient identifiers are not available for NDRI-supplied globes because there are written policies and procedures prohibiting the release of this information. Upon arrival, globes were opened and the RPE cells were harvested and maintained as previously described; these spontaneously immortalized cells from passage 4 were placed in medium containing 10% dimethyl sulfoxide (DMSO) and frozen in liquid N_2_ until use (Jun et al. 2017; Calandria et al. 2012).

### PC and Oxidative Stress

Cells were plated in 48-well plates (10,000 cells/well, 10% fetal bovine serum [FBS] DMEM/D12 1:1 medium), grown to confluency (72 h), and placed in 1% FBS for 18 h prior to experimental treatment. Cells were then stimulated with 50 µM H_2_O_2_ (2 h half-life) for 6 h (PC). The medium was then removed, and the cells placed back in 1% FBS medium for 24 h. In order to ensure that we were able to observe 30–70% cell death in the given cell passage or hydrogen peroxide potency, cells were stressed using increasing concentrations of oxidative stress (OS; 300–800 µM H_2_O_2_) plus 10 ng/ml TNFα for 16 h. It is important to note that the amount of OS necessary to cause nuclear pyknosis positively correlated with increases in the cell passage (electronic supplementary Fig. 2). Cell survival was quantified by nuclear pyknosis using the morphonuclear analysis imaging method (MAIM), as previously verified and described by Stark and Bazan 2011. Only wells with OS control conditions indicating 30–70% cell death are reported in this study. Media and cells were also collected at various time points for lipid analysis via ultra-performance liquid chromatography-electrospray ionization-tandem mass spectrometry (UPLC-ESI-MS/MS) and protein analysis.

### Treatment and Co-treatment

Human RPE cells were grown as mentioned above. After 18 h in 1% FBS medium, cells were treated with DHA, NPD1, PEDF, or PEDF fragments for 6 h then exposed to PC for 6 h. The medium was then removed and the cells were allowed to rest for 24 h in 1% FBS medium, and then subsequently stressed with 300–800 µM H_2_O_2_ plus 10 ng/ml TNFα for 16 h. The MAIM was used to quantify cell survival (Stark and Bazan 2011).

### Spectral Domain-Optical Coherence Tomography

Male Sprague–Dawley rats (150–175 g) were imaged before and after experimentation by SD-OCT. Animals were anesthetized with ketamine (35 mg/kg; Parke-Davis, Morris Plains, NJ, USA), and xylazine (5 mg/kg; Miles, Shawnee Mission, KS, USA). The eyes were then dilated with tropicamide ophthalmic solution (1%; Akorn Pharmaceticals, Lake Forest, IL, USA), and small drops of methyl cellulose (1%) were placed on the eyes to prevent corneal desiccation. As previously described by Knott et al. 2011, retinas were imaged by SD-OCT along the vertical meridian through the optic disc (superior retina, optic nerve, inferior retina) using a Heidelberg Spectralis HRA + OCT system (Heidelberg Engineering, Heidelberg, Germany). Signal quality was >20 db, and scan speed was 40,000 A-scans per second (at least 25 B-scan frames were averaged). Axial resolution was 7 mm optical and 3.5 mm digital. Animals were then allowed to recover in their cages and were returned to the LSU Health New Orleans animal care facility.

### Ultra-Performance Liquid Chromatography-Electrospray Ionization-Tandem Mass Spectrometry

Lipidomic experiments were conducted using human cells in 10 cm culture plates (2 million cells/well; 10 ml of stimulation medium/well; 1 plate/sample) or retinal tissue from rat retinas. Samples were collected, homogenized, and sonicated with 100% methanol (MeOH) plus butylated hydroxytoluene (BHT, 1 g/l), and internal standards were added (prostaglandin [PG] D2-deuterated, PGE2-deuterated, 15-hydroxyeicosatetraenoic acid (HETE)-D8, DHA-D5, and AA-D8; Cayman Chemicals, Ann Arbor, MI, USA). Samples were then stored at −80 °C overnight and centrifuged at 4200 RCF for 30 min. Supernatants were diluted to <10% MeOH, acidified (pH ≤3.5), and applied to pre-equilibrated columns (BondElut C18 solid-phase extraction columns; Agilent, Santa Clara, CA, USA). Columns were sequentially rinsed with 10 ml of water (pH 3.5) and 1 ml of hexane. Target compounds were eluted with methyl formate, and the eluent was dried under nitrogen. Extracts were resuspended in 200 μl MeOH, transferred to vials, redried under nitrogen, and resuspended in 12 µl MeOH and 3 µl water (pH 7). Extracts (10 μl injection volumes) were injected into a Xevo TSQ triple quadrupole mass spectrometer (Waters, Boston, MA, USA) equipped with an electrospray ionization probe (operated in the negative ion mode) and Acquity I-Class Sample Manager/combined UPLC pump running a mobile phase of methanol:water 70/30%. The instrument was operated in full scan mode, as indicated. Since not all target compounds have a commercially available deuterium standard, target compounds were normalized to the internal standards listed above and quantified using a standardized concentration curve. Total sample concentrations were normalized to total protein content as determined by a Bradford assay.

### Western Blot Analysis

Media were collected and concentrated using Amicon Ultra 0.5 ml centrifugal filters (Millipore), and cells from in vitro experiments were homogenized in a radioimmunoprecipitation (RIPA) lysis buffer. Protein concentrations were determined by bicinchoninic acid (BCA) assay (Bio Rad, Hercules, CA, USA). Aliquots of proteins from cells or medium were loaded into a 4–12% polyacrylamide gel (Invitrogen, Carlsbad, CA, USA) and electrophoresed on ice at 125 volts for 2 h. Proteins were transferred to a polyvinylidene difluoride (PVDF) membrane via an iBlot transfer system (Invitrogen, Carlsbad CA, USA). Western blot analysis was performed on media for the neurotrophin PEDF (anti-PDEF, 1:1000; Santa Cruz, San Diego, CA, USA) and in cell extracts for phosphorylated ERK 1/2 (anti-p-ERK 1/2, 1:500; Millipore, Billerica, MA) expression. The blot was stripped and probed with β-actin (anti-β actin, 1:10,000; Sigma, St. Louis, MO, USA), and p-ERK and PEDF expression levels were normalized to β-actin (anti-β actin, 1:10,000; Sigma, St. Louis, MO, USA). Secondary horseradish peroxidase antibodies were exposed with Amersham ECL Western blotting detection on a Fuji scanner LAS 4000.

### Statistical Analysis

All in vitro and in vivo assessments were performed in a double-blind manner. Data are expressed as mean ± standard error of the mean. Statistical analysis was carried out using Student’s *t*-test or a one-way analysis of variance (ANOVA), followed by either a Tukey test (balanced sample numbers) or a Dunnett *t*-test (unbalanced sample numbers) [* *p* ≤ 0.05 and # *p* ≤ 0.001].

## Results

### PC Protection Increases Unesterified Docosahexaenoic Acid (DHA) and Arachidonic Acid (AA) Pool Sizes and Stimulates Pigment Epithelium-Derived Factor (PEDF) Synthesis

**Fig. 1:**
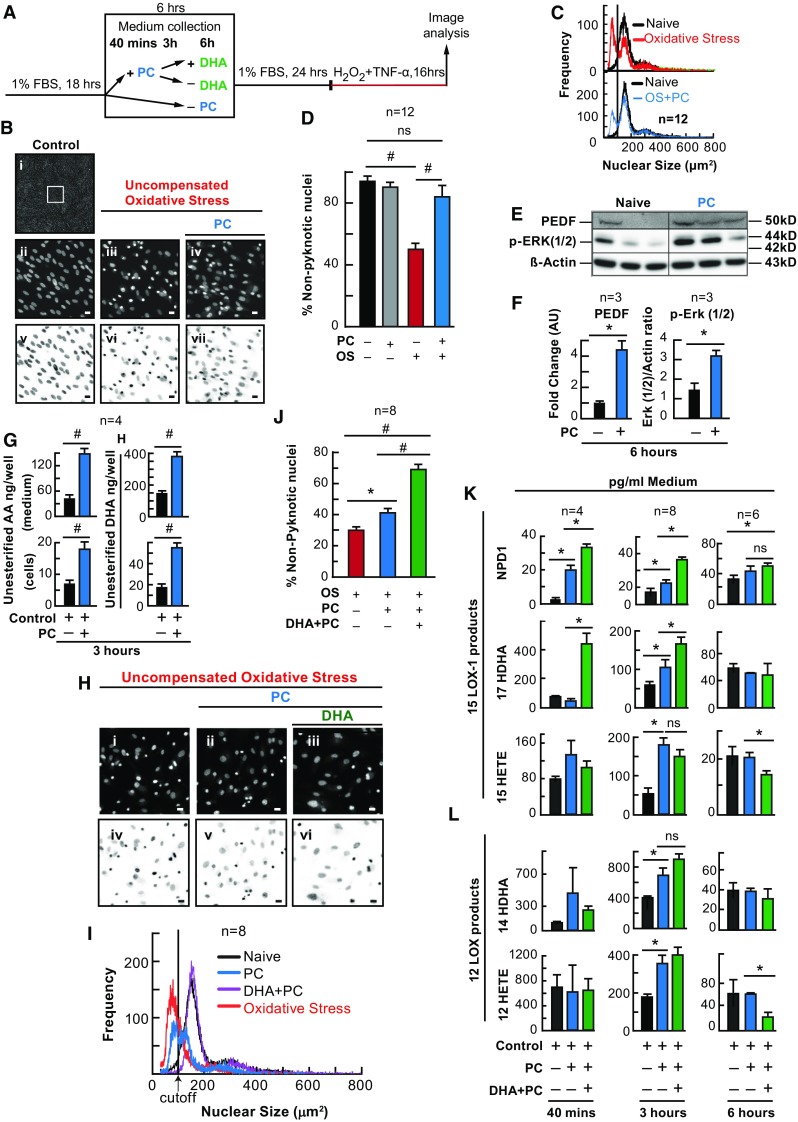
Docosahexaenoic acid enhances PC protection. APRE-19 cells were treated with DHA or vehicle for 6 h prior to mild oxidative preconditioning and subsequent lethal OS. Cell and media were collected for protein and lipid analysis and nuclei were imaged for cell survival using MAIM. **a** Experimental design. **b** (*i*) Example 8-bit grey-scale confocal image (10× magnification), zoomed-in (**b** *ii*–*iv* and **h** *i*–*iii*), and inverted micrographs (**b** *v*–*vii* and **h** *iv*–*vi*) of human ARPE-19 cell nuclei stained with Hoestch 33258. **c**, **i** Nuclear size frequency histogram plots of graphs **d** and **j**. **d**, **j** Quantification of frequency plots as a percentage of non-nuclear pyknosis (% survival) in cells preconditioned with ±50 µM H_2_O_2_ and/or DHA (200 nM) for 6 h. **e** Western blot of pigment PEDF (50 kDa); phosphorylated ERK 1 and 2 (44 and 42 kDa) and β-actin as the loading control (43 kDa) following 6 h of PC. **f** Western blot ratio quantification normalized to total β-actin. **g** Quantification of non-esterified DHA and AA in cells and media of ARPE-19 cells treated with ±50 µM H_2_O_2_ for 3 h. **k** UPLC-ESI-MS/MS quantification of 15-LOX-1 products of DHA and AA, NPD1, 17-HDHA, and 15-HETE at 40 min, 3 h and 6 h of PC. **l** Quantification of 12 LOX products of DHA and AA, 12-HETE and 14-HDHA at 40 min, 3 h and 6 h of PC. All lipid concentrations were normalized to total protein determined by Bradford assay or media volume. *p < 0.05, ^#^ *p* < 0.001, scale bar 10 micro molar (µM). *ERK* extracellular signal-regulated kinases

Sublethal concentrations of hydrogen peroxide 50 µM H_2_O_2_ (a mild oxidative PC-stimulus) have been shown to subsequently protect RPE cells by preventing cellular detachment (Sharma et al. 2009). We have shown that DHA is released in response to OS and protects ARPE-19 cells (Mukherjee et al. 2004). Therefore, we wanted to determine if 50 µM H_2_O_2_ (PC-stimulus) could reduce nuclear pyknosis in ARPE-19 cells lethally challenged with OS, and if the endogenous non-esterified DHA pool was concomitantly increased in response to PC-stimulus or during PC. To test this, we stimulated human ARPE-19 cells with 50 µM H_2_O_2_ 24 h prior to a lethal oxidative challenge (Fig. 1a), and cell death was assessed using a verified, automated, non-biased, cell counting method, MAIM (Stark and Bazan 2011). It was found that cultures treated with lethal OS had an increase in cell death signified by increased nuclear pyknosis (*p* ≤ 0.001, ANOVA/Tukey), while cultures previously exposed to PC stimulus were protected from this increase (*p* ≤ 0.001, ANOVA/Tukey) (Fig. 1b–d). UPLC-ESI–MS/MS lipid analysis revealed that there was an increase in the pool of DHA and AA in both cells (*p* ≤ 0.001, *p* ≤ 0.001) and the media (*p* ≤ 0.001, *p* ≤ 0.001) following 3 h of the PC stimulus (Fig. 1g) [Student’s *t*-test]. Furthermore, it was also revealed that the neurotrophin, PEDF, was increased in the media of these cells 6 h after the PC stimulus (*p* ≤ 0.05, Student’s *t*-test), which was evident with concomitant cell expression of phosphorylated ERK 1 and 2 expression (Fig. 1e, f) [*p* ≤ 0.05, Student’s *t*-test].

### DHA Enhances PC Protection and Alters Eicosanoid and Neuroprotectin D1 (NPD1) Synthesis in Primary Human RPE Cells

**Fig. 2:**
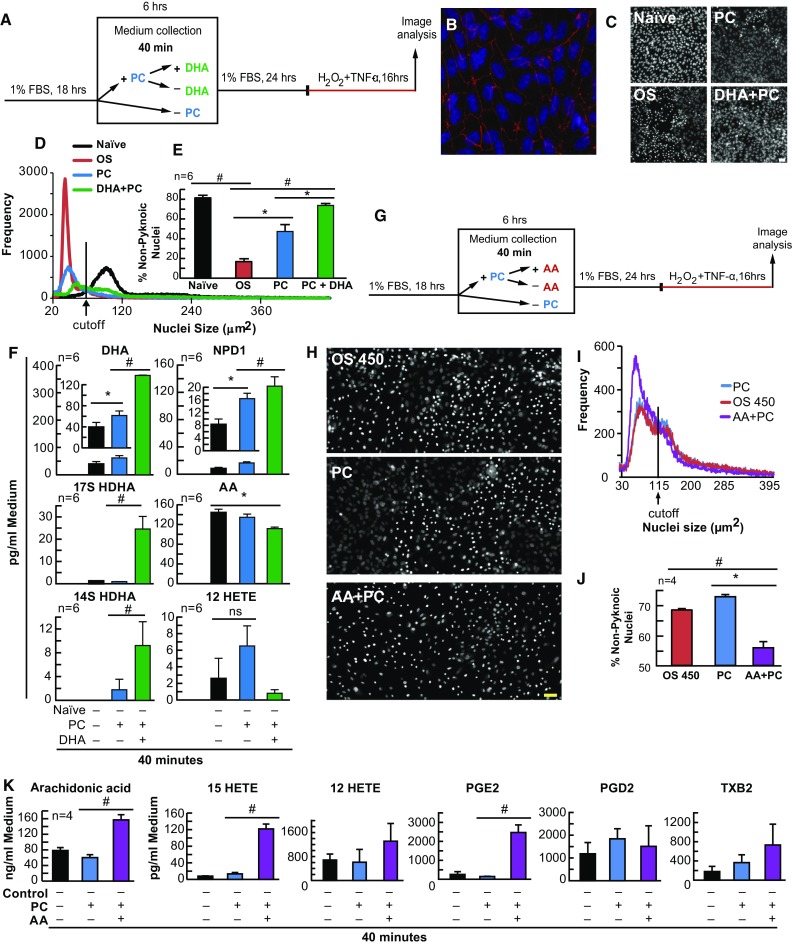
DHA enhances and AA attenuates PC in human RPE cells in vitro. APRE-19 and human primary cells were treated with DHA, AA, or vehicle 6 h prior to mild oxidative preconditioning and lethal OS. Medium was collected for lipid analysis and nuclei were imaged for cell survival using MAIM. **a** Experimental design illustration for DHA using primary human RPE cells. The following are examples of the characterization of these cell responses: **b** Confocal image 20× magnification and zoomed-in human primary RPE cells stained with Hoestch 33258 and probed for ZO-1; **c** 8-bit grey-scale confocal image (20× magnification) of human primary RPE cells stained with Hoestch 33258; and **d** nuclear size frequency histogram plots for PC + DHA. **e** Quantification of frequency plots as a percentage of non-nuclear pyknosis (% survival) in cells preconditioned with ±50 µM H_2_O_2_ and/or DHA (200 nM) for 6 h prior to lethal OS challenge. **f** UPLC-ESI-MS/MS quantification of AA, DHA, and derivatives 12-HETE, 17-HDHA, 14-HDHA, and NPD1 at 40 min PC and PC + 200 nM DHA. **g** Experimental design for AA and ARPE-19 cells. The following are examples of the characterization of these cell responses: **h** 8-bit grey-scale confocal image (20× magnification) of ARPE-19 cells stained with Hoestch 33258; and **i** nuclear size frequency histogram plot for PC + AA. **j** Quantification of frequency plots as a percentage of non-nuclear pyknosis (% survival) in cells preconditioned with ±50 µM H_2_O_2_ and/or 200 nM AA for 6 h prior to lethal OS challenge. **k** UPLC-ESI-MS/MS quantification of AA and derivatives 15-HETE, 12-HETE, PGE2, PGD2 and TXB2 at 40 min PC and PC + 200 nM AA. All lipid concentrations were normalized using deuterium-labeled internal standards and to medium volume. *p < 0.05, ^#^ *p* < 0.001, scale bar 10 µM

Since DHA is released during PC, and DHA as well as its metabolic derivatives have been shown to be protective, we wanted to examine whether DHA would enhance the protection bestowed by PC stimulus and whether a previous DHA supplementation would alter lipoxygenase-mediated eicosanoid and docosanoid synthesis. ARPE-19 (Fig. 1h–l) and human primary RPE cells (Fig. 2) were exposed to PC-stimulus, with and without a previous 6-h treatment of DHA (200 nM), followed by lethal OS. When exposed to PC-stimulus, both ARPE-19 (*p* ≤ 0.05, ANOVA-Tukey) and human primary RPE cells (*p* ≤ 0.05, ANOVA-Tukey) became resistant to lethal OS, and when cells were supplemented with DHA (200 nM) 6 h prior to PC-stimulus (not during), the protection was enhanced (Fig. 1h, i [*p* ≤ 0.001, ANOVA-Tukey], and Fig. 2b–e [*p* ≤ 0.001, ANOVA-Tukey]). In media extracts, both eicosanoids and docosanoids were synthesized during PC. However, when DHA was supplemented to ARPE-19 cell cultures prior to PC-stimulus, docosanoid (17-hydroxy docosahexaenoic acid [17-HDHA] and NPD1) synthesis increased (*p* ≤ 0.05, ANOVA-Tukey) at 40 min and 3 h, while eicosanoid (12- and 15-HETE) synthesis increased at 3 h (*p* ≤ 0.05, ANOVA-Tukey), but was decreased at 6 h (*p* ≤ 0.05, ANOVA-Tukey) (Figs. 1k and 2f). In human primary RPE cells, NDP1 increased at 40 min (*p* ≤ 0.05, ANOVA-Tukey) during PC, while 17-HDHA (*p* ≤ 0.05, ANOVA-Tukey) only increased during DHA supplementation.

### AA Attenuates PC Protection

It has been previously reported that AA and its metabolites are necessary for myocardial IPC (Murphy et al. 1995). Since AA is released and eicosanoids are synthesized in response to PC-stimulus or during PC in RPE cells, we wanted to determine whether the enhanced protection displayed was specific to DHA. Therefore, we examined whether AA supplementation 6 h prior to PC-stimulation would elicit the same enhanced protective effect. ARPE-19 cells were treated with PC-stimulus, with and without a prior 6-h treatment of AA (200 nM), and the media were collected for UPLC-ESI-MS/MS analysis at 40 min (Fig. 2g). As before, when cells were exposed to PC-stimulus, there was a trending, however not initially significant, reduction in nuclear pyknosis (protection; *p* = 0.091, ANOVA-Tukey). However, when 200 nM of AA was supplemented 6 h prior to PC stimulus, there was a dramatic decrease in non-pyknotic nuclei (Fig. 2h–j) [*p* ≤ 0.001, ANOVA-Tukey]. Furthermore, lipid media extracts indicated that the AA-derived eicosanoid products 15-HETE and PGE2 were increased when exogenous AA was supplemented (Fig. 2k) [*p* ≤ 0.001, ANOVA-Tukey; and *p* ≤ 0.001, ANOVA-Tukey, respectively].

### NPD1 and PEDF Enhance PC Protection, While PEDF and its Fragments Stimulate 17-Hydroxy Docosahexaenoic Acid (17-HDHA) and NPD1 Synthesis

**Fig. 3:**
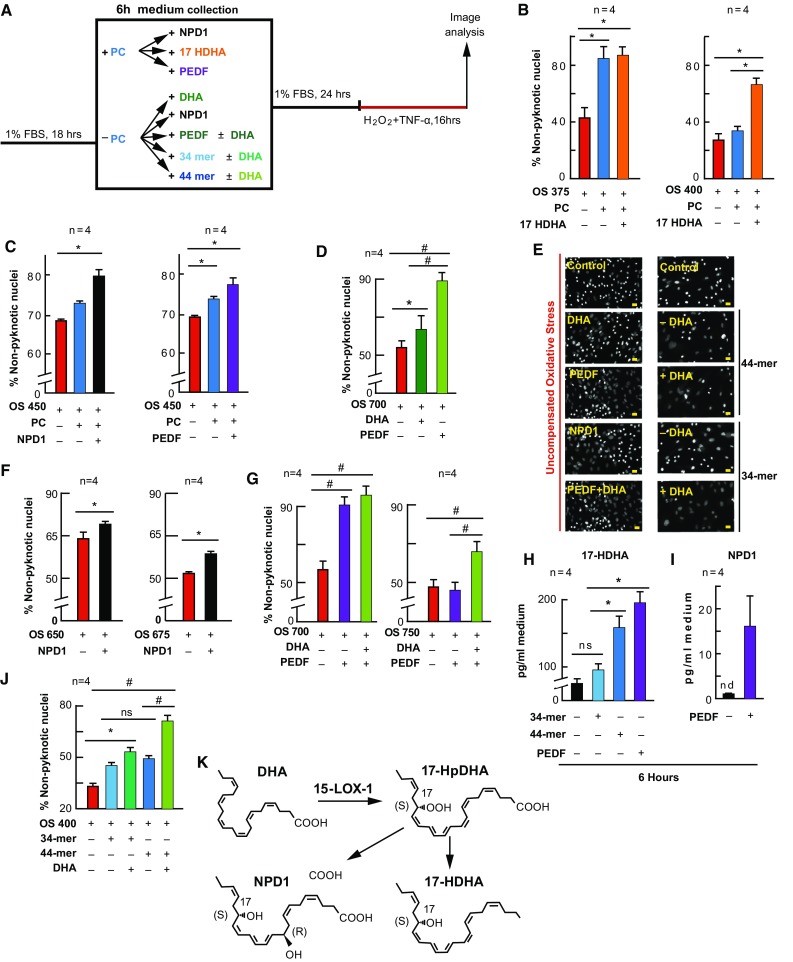
NPD1 and PEDF enhance and mimic PC. APRE-19 cells were treated with NPD1, 17-HDHA, PEDF, or vehicle ± mild oxidative preconditioning prior to lethal OS. Cell nuclei were imaged for cell survival using MAIM, and media was collected for lipid analysis after 6 h PEDF or PEDF fragment supplementation. **a** Experimental design. **b**, **c**, **d**, **f**, **g**, **j** Quantification of non-nuclear pyknosis (protection) ±50 µM H_2_O_2_, DHA (200 nM), NPD1 (100 nM), 17-HDHA (100 nM), PEDF (1 nM), or PEDF protein fragments 34- or 44-mer. **e** Example 10×, zoomed-in grey-scale micrographs of nuclei treated with 17-HDHA, NPD1, PEDF, 34-mer, or 44-mer ± 200 nM DHA, and stained with Hoestch 33258. **k** Schematic depicting DHA conversion to 17-HDHA and NPD1 via 15-LOX-1. **h**, **i** UPLC-ESI-MS/MS quantification of 17-HDHA and NPD1 following 6 h supplementation of PEDF, 34-mer, or 44 mer. Lipid concentrations were normalized using deuterium-labeled internal standards and to medium volume. * *p* < 0.05, ^#^ *p* < 0.001), scale bar 10 µM

We determined that non-esterified DHA and PEDF were increased, 17-HDHA and NPD1 were synthesized, and PC was enhanced by DHA. Therefore, we wanted to examine whether supplementing PEDF, 17-HDHA, or NPD1 would also enhance PC protection. To accomplish this, each compound was supplemented to cells for 6 h prior to PC-stimulation (Fig. 3a; electronic supplementary Fig. 3). Following treatment periods and PC, media were collected and replaced with fresh media for 24 h. Cells were then challenged lethally with OS, and cell death was assessed via MAIM. When cells were treated with PC-stimulus previously supplemented with NPD1 (100 nM; *p* ≤ 0.05, ANOVA-Tukey), 17-HDHA (100 nM; *p* ≤ 0.05, ANOVA-Tukey), and PEDF (1 nM; *p* ≤ 0.05, ANOVA-Tukey), there was a significant increase in the number of non-pyknotic nuclei (protection) when compared with uncompensated OS alone and PC plus uncompensated OS (Fig. 3b; electronic supplementary Fig. 3a, d, g) [*p* ≤ 0.05, ANOVA-Tukey].

### NPD1 and 17-HDHA Enhance, While DHA, NPD1, PEDF, and Fragments Mimic, PC Protection

If DHA, PEDF, 17-HDHA and NPD1 are players in the protective actions of PC, it would be reasonable to assume that they could enhance PC protection and also stimulate a protective action without the presence of the PC-stimulus (50 µM H_2_O_2_) (Fig. 3). Therefore, we supplemented 17-HDHA, NPD1, and PEDF to cultures for 6 h prior to the PC stimulus, and DHA, NPD1, PEDF, and PEDF fragments without treating them with the PC stimulus. The treatment medium was removed for 24 h under both paradigms, and the cultures were subsequently challenged with lethal OS (Fig. 3a). As shown in Figs. 1 and 2, when cells were stimulated with PC-stimulus there was an increase in non-pyknotic nuclei. When 17-HDHA (*p* ≤ 0.05, ANOVA-Tukey) and NPD1 (*p* ≤ 0.05, ANOVA-Tukey) were added to the cultures of increasing lethal OS concentrations, this resulted in a further increase in protection (*p* ≤ 0.05, ANOVA-Tukey; and *p* ≤ 0.05, ANOVA-Tukey, respectively) (Fig. 3b–d). When DHA (*p* ≤ 0.05, ANOVA-Tukey), NPD1(*p* ≤ 0.05, Student’s *t*-test), PEDF (*p* ≤ 0.05, ANOVA-Tukey) and 34- and 44-mer fragments of PEDF (*p* ≤ 0.05, ANOVA-Tukey; and *p* ≤ 0.05, ANOVA-Tukey, respectively) were added to the treatment media for 6 h without PC-stimulus and allowed to recover for 24 h, these cells were also protected from lethal OS (Fig. 3f, g, j). When DHA was added to cultures in combination with PEDF or 34- and 44-mer fragments of PEDF, the effect was greater than when either DHA (*p* ≤ 0.05, ANOVA-Tukey) or PEDF (*p* ≤ 0.05, ANOVA-Tukey) were alone (Fig. 3g, j; electronic supplementary Fig. 3c) [ANOVA-Tukey]. Moreover, media collected from PEDF and 44-mer fragment supplementation demonstrated an increase in the synthesis of 17-HDHA (*p* ≤ 0.05, ANOVA-Tukey) (Fig. 3h); however, only PEDF was able to trigger NPD1 synthesis under these conditions (Fig. 3i).

### PD-146176 Exacerbates Cell Death and Blocks PEDF Protection by Inhibition of 17-HDHA, NPD1, and 15-Hydroxyeicosatetraenoic Acid (HETE) Synthesis

**Fig. 4:**
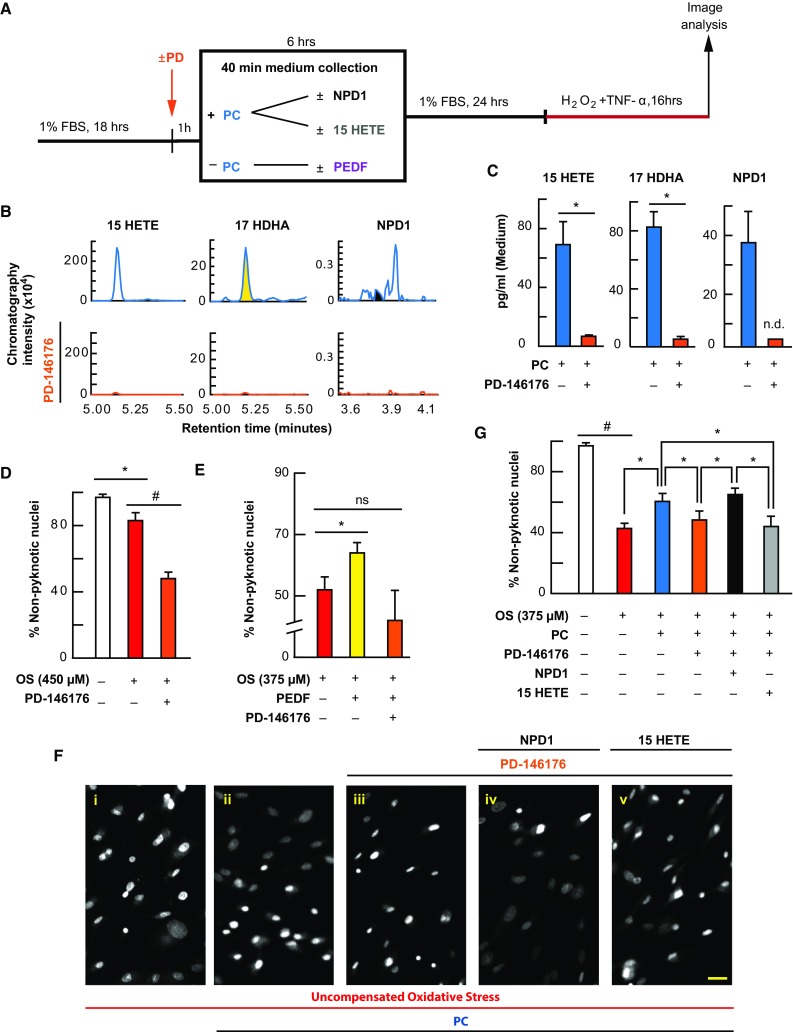
PC protection is blocked by 15-LOX-1 inhibition and is selectively rescued by exogenous NPD1. APRE-19 cells were treated with vehicle, PD-146176, NPD1, 15-HETE, or PEDF prior to mild oxidative preconditioning and lethal OS. Cell and media were collected for lipid analysis, and nuclei were imaged for cell survival using MAIM. **a** Experimental design. **b** Typical mass spectrometry chromatograms of 15-HETE, 17-HDHA, and NPD1 during PC with and without the 15-LOX-1 inhibitor PD-146176. **c** UPLC-ESI-MS/MS graphical quantification of 15-HETE, 17-HDHA, and NPD1 during PC with and without PD-146176. **d**, **e** Quantification of percentage of non-pyknotic nuclei challenged with lower concentrations of uncompensated OS with and without PD-146176 (2 µM) or PEDF (1 nM) with and without PD-146176 for 6 h prior to lethal challenge. **f** Representative 8-bit 10× zoomed-in micrographs of ARPE cells treated ± PC ± PD-146176 in combination with 100 nM 15-HETE or 100 nM NPD1. **g** Graphical quantification of percentage of non-pyknotic nuclei during lethal oxidative challenge with and without PC, PD-146176, NPD1, or 15-HETE. *p < 0.05, ^#^ *p* < 0.001, scale bar 10 µM

We have previously shown that the stable knockdown of 15-LOX-1 by short hairpin RNA (shRNA) results in increased sensitivity to OS, while 17-HDHA, 15-HETE, and NPD1 are diminished (Calandria et al. 2009). It has been demonstrated that PD-146176 is specific to 15-LOX-1 and does not affect 12- or 15-LOX activity (Nair and Funk 2009). Therefore, to explore whether 15-LOX-1 is necessary for the protective actions taking place in RPE cells, we treated these cells with PD-146176 (2 µM) 1 h prior to and during lethal OS and 6-h PEDF (1 nM) pretreatment (Fig. 4a). While low concentrations of OS resulted in a decrease in non-pyknotic nuclei (*p* ≤ 0.05, ANOVA-Tukey), PD-146176 exacerbated the cell death of the lethally-challenged APRE-19 cells (Fig. 4d) [*p* ≤ 0.001, ANOVA-Tukey] and attenuated PEDF pretreatment protection (Fig. 4e; electronic supplementary Fig. 3e) [*p* ≤ 0.05, ANOVA-Tukey], while attenuating production of 17-HDHA (*p* ≤ 0.05, Student’s *t*-test), 15-HETE (*p* ≤ 0.05, Student’s *t*-test), and NPD1 (non-significant) (Fig. 4b, c).

### NPD1 Selectively Rescues PD-16176 Inhibition of PC Protection

It has been previously demonstrated that broad spectrum inhibition of lipoxygenase activity eliminates the protective actions of ischemic cardiac PC (Murphy et al. 1995). Since the AA product of 15-LOX-1 (15-HETE) is generated during PC and is inhibited by PD-146176, it was necessary to differentiate the activity of some of the docosanoids and eicosanoids. Therefore, we treated ARPE-19 cells with PD-146176 (2 µM) 1 h prior to and during PC stimulus, with and without NPD1 (100 nM) and 15-HETE (100 nM). After 6 h, media were removed and cells were allowed to rest for 24 h prior to being subjected to lethal OS. When cells were challenged lethally, nuclear pyknosis increased (*p* ≤ 0.001, ANOVA-Tukey). When cells were treated with PC-stimulus, significant protection occurred (*p* ≤ 0.05, ANOVA-Tukey), and when cells were treated with PD-146176 1 h prior to and during PC, cells had less non-pyknotic nuclei when compared with PC-stimulated cells (*p* ≤ 0.05, ANOVA-Tukey). However, when NPD1 and 15-HETE were added concomitantly with PD-146176 during PC, NPD1 selectively increased the number of non-pyknotic nuclei (*p* ≤ 0.05, ANOVA-Tukey) when compared with PD-146176-treated cells, while 15-HETE was unable to elicit the same protective effect when compared with PD-146176-treated cells (*p* = 0.489, ANOVA-Tukey) (Fig. 4f, g; electronic supplementary Fig. 3h).

### IPC Requires Docosanoid and Eicosanoid Signaling

**Fig. 5:**
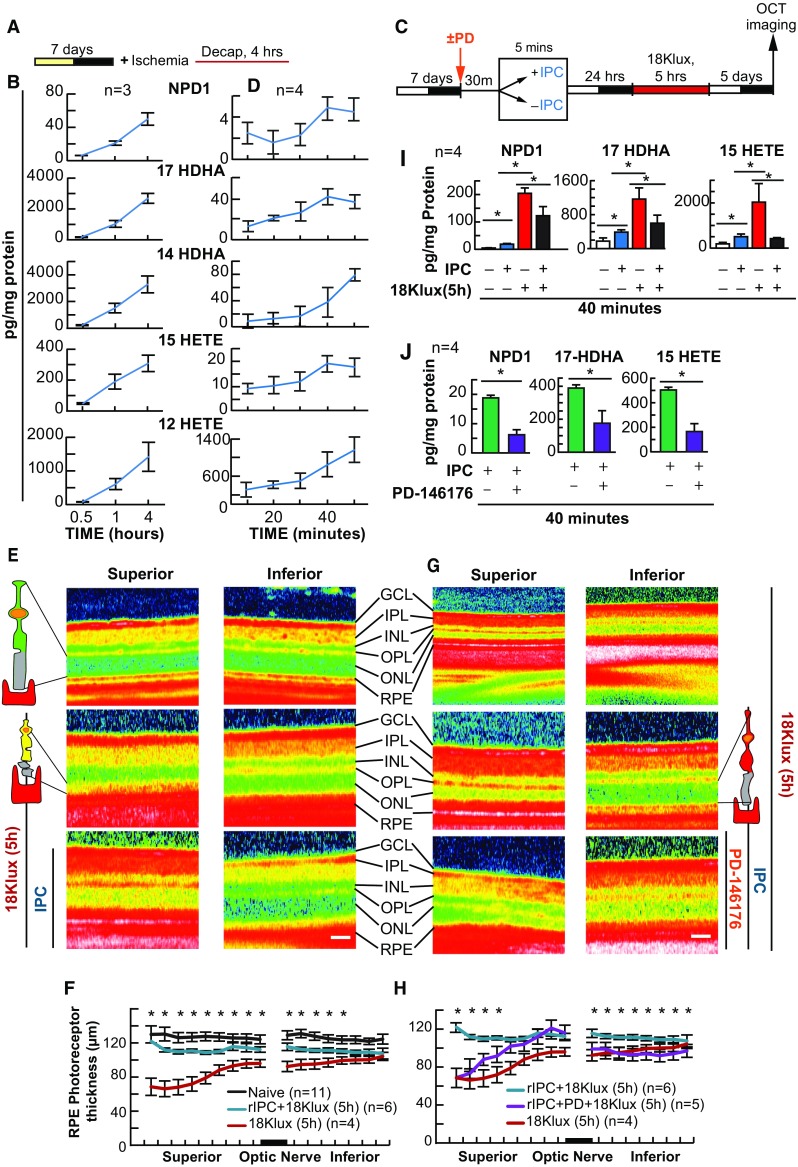
IPC triggers NPD1 and eicosanoid synthesis, and is blocked by PD-146176. Mice were subjected to elevated intraocular pressure ± PD-146176 for 5 min, 24 h prior to exposure to lethal bright light. Outer retinal thickness was measured and retinas collected for lipid analysis. **a**, **c** Experimental design of post-decapitation ischemia and 5 min hydrostatic-induced ischemic PC ± lethal light exposure. **b** UPLC-ESI-MS/MS graphical quantification of NPD1 and eicosanoid abundance post decapitation-induced ischemia. **d** 5-min hydrostatic pressure-induced ischemia. **e**, **g** Representative SD-OCT images of retina exposed to 5 min of ischemia or control conditions prior to lethal light exposure. **f**, **h** Outer retinal thickness measurements following light damage ± IPC and/or PD-146176. **i** IPC ± lethal light exposure. **j** IPC ± PD-146176. *p < 0.05, ^#^ *p* < 0.001, scale bar 120 µM. *IPC* ischemic retinal preconditioning

Five minutes of ischemia protects cardiac myocytes from lethal myocardial ischemia, while non-esterified DHA and AA pools are increased (Murphy et al. 1995). Furthermore, 5 min of increased IOP-induced ischemia also protects photoreceptors from bright light-induced retinal degeneration (Casson et al. 2003). Since we have determined that docosanoid synthesis is pivotal in RPE-PC, we wanted to test the hypothesis that this mechanism involving DHA and docosanoids is conserved in an in vivo model of retinal IPC; however, before we could test this hypothesis, we first needed to confirm that rat retinas could synthesize docosanoids and eicosanoids under ischemic conditions. Retinas of animals subjected to chronic ischemia via decapitation resulted in a temporal increase in NPD1, 17-HDHA, 14-HDHA, 15-HETE, and 12-HETE (*p* ≤ 0.05 when compared with the 30-min time point, ANOVA-Dunnett *t*-test) (Fig. 5a, b). Following this result, rat retinas were exposed to IPC and collected at 10-min intervals to determine the appropriate collection time for lipid analysis. The resulting data showed that there was a temporal increase in both eicosanoids 12-HETE (*p* ≤ 0.05, Student’s *t*-test) and 15-HETE (*p* ≤ 0.05, Student’s *t*-test) and docosanoids NPD1 (*p* ≤ 0.05, Student’s *t*-test), 17 HDHA (*p* ≤ 0.05, Student’s *t*-test), and 14-HDHA (*p* ≤ 0.05, Student’s *t*-test), which peaked around 40 min after IPC reperfusion when compared with baseline (10 min) (Fig. 5c, d). As expected, when rats were exposed to lethal bright light (18 kLux) for 5 h, when compared with dark controls, there was a significant decrease in outer retinal thickness (photoreceptor terminal to Bruch’s membrane) [*p* ≤ 0.05, Student’s *t*-test], and prior exposure of IPC significantly reduced the effect of bright light exposure (*p* ≤ 0.05, Student’s *t*-test) (Fig. 5e, f). However, treatment with the 15-LOX-1 inhibitor PD-146176 30 min before and during IPC was able to eliminate the observed protective effects of IPC (*p* ≤ 0.05, Student’s *t*-test) (Fig. 5g, h, j). Moreover, UPLC-ESI-MS/MS lipid analysis (40 min) of retinas exposed to IPC revealed the triggering of NPD1 (*p* ≤ 0.05, Student’s *t*-test), 17-HDHA (*p* ≤ 0.05, Student’s *t*-test), and 15-HETE (*p* ≤ 0.05, Student’s *t*-test) synthesis when compared with dark controls (Fig. 5i); however, if retinas were injected with PD-146176, this increase was diminished (Fig. 5j). Bright light also stimulated synthesis of NPD1 (*p* ≤ 0.05, Student’s *t*-test), 17-HDHA (*p* ≤ 0.05, Student’s *t*-test), and 15-HETE (*p* ≤ 0.05, Student’s *t*-test) (Fig. 5i), but if retinas were exposed to IPC 24 h prior to bright light exposure, this increase in NPD1 (*p* ≤ 0.05, Student’s *t*-test), 17-HDHA, (*p* ≤ 0.05, Student’s *t*-test), and 15-HETE (*p* ≤ 0.05, Student’s *t*-test) that was seen in bright light was blunted (*p* ≤ 0.05, Student’s *t*-test) (Fig. 5i).

### LPC Protection Triggers 17-HDHA and NPD1 Synthesis

**Fig. 6:**
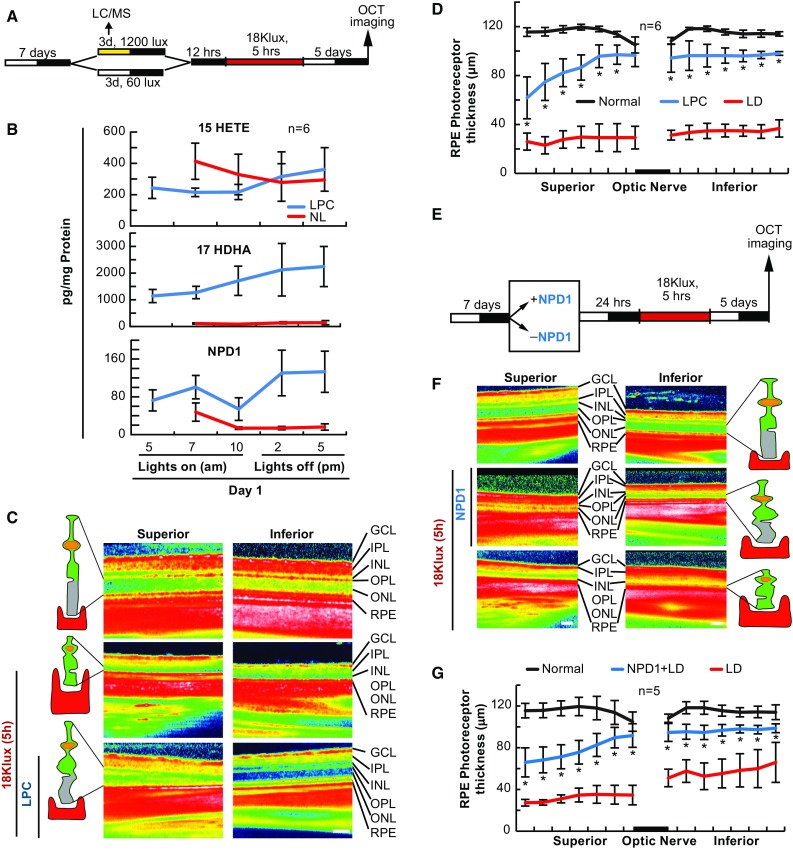
NPD1 protects photoreceptor cells from bright light-induced degeneration. Mice were subjected to moderate and normal light for 3 days, or injected with NPD1, 24 h prior to exposure to lethal bright light. Outer retinal thickness was measured and retinas collected for lipid analysis. **a**, **e** Experimental designs. **b** UPLC-ESI-MS/MS graphical quantification of normal light and light preconditioning for 15-HETE, 17-HDHA, and NPD1. **c**, **f** Representative SD-OCT images of retinas treated with 40 lux ambient light or 1200 lux for 3 days prior to lethal light exposure or exposed to NPD1 100 ng prior to lethal light exposure. (**d**) Graphical quantification of outer retinal thickness measurements following dark, LPC + LD or dark + LD. **g** Outer retinal thickness measurements following LD ± 100 ng NPD1. *p < 0.05, ^#^ *p* < 0.001, scale bar 120 µM. *LD* light damage

Moderate light exposure protects photoreceptors from bright light-induced photoreceptor degeneration (Liu et al. 1998). Since PC of RPE cells relies on docosanoid signaling cascades, and since IPC requires 15-LOX-1 products, we wanted to determine if light-stimulated PC increased docosanoid synthesis. To test this, albino rats were exposed to moderate light (LPC, 1200 Lux, 12 h on/12 h off) or normal light (NL; 60 Lux, 12 h on/12 h off) for 3 days prior to lethal bright light exposure (Fig. 6a). As expected, when exposed to 3 days of ambient light and 5 h of bright light, there was an approximate 50% reduction in outer retinal thickness when compared with dark controls (*p* ≤ 0.05, Student’s *t*-test). However, when animals were exposed to LPC prior to bright light exposure, photoreceptors were significantly protected from this degeneration (*p* ≤ 0.05, Student’s *t*-test) (Fig. 6c, d). UPLC-ESI-MS/MS analysis at various times throughout the first day revealed that 17-HDHA and NPD1 were stimulated by LPC, while 15-HETE was not differentially regulated when compared with NL-cycled animals (*p* = 0.842, Student’s *t*-test) (Fig. 6b).

### NPD1 Rescues Photoreceptors from Bright Light-Induced Degeneration

We have shown previously that NPD1 protects RPE cells from cell death in response to lethal OS and ischemia reperfusion (Mukherjee et al. 2007; Bazan et al. 2011a, b), and that stable knockdown of NPD1 synthesis increases RPE cell sensitivity to OS (Calandria and Bazan 2010). Since NPD1 is increased during the in vivo IPC resolution phase and LPC stimulus phase, and is required for RPE cell PC, we wanted to determine whether NPD1 could protect photoreceptors from bright light-induced degeneration. To do this, NPD1 (100 ng) was injected into the anterior chamber 1 h prior to lethal bright light. In the retinas where NPD1 was not added, the bright light induced outer retinal degeneration, as expected; however, when the 100 ng of NPD1 was injected into the anterior chamber 1 h prior to bright light exposure, NPD1 significantly protected the outer retina from degeneration (*p* ≤ 0.05, Student’s *t*-test for each point) (Fig. 6e–g).

## Discussion

DHA and its metabolic derivatives docosanoids demonstrate potent protective, anti-inflammatory, and pro-resolution bioactivities (Bazan et al. 2010; Calandria and Bazan 2010; Cortina et al. 2010). Previous reports have shown that DHA has positive effects on patients with dry-eye disease and age-related macular degeneration (Chung et al. 2008; Tanito et al. 2010). Thus, understanding the underlying role of DHA and docosanoids during retinal PC may lead to advanced treatments for many retinal degenerative diseases.

In this study, we show for the first time that the protective actions of oxidative PC require DHA and docosanoid synthesis, while also showing that DHA and docosanoids are involved in both IPC and LPC protection. In RPE PC, there is an increase in DHA and AA pool size with concomitant eicosanoid and docosanoid synthesis, and we demonstrate that DHA and the docosanoids 17-HDHA and NPD1 enhance and mimic the effects of this PC protection, while AA does not. Furthermore, inhibition of 15-LOX-1 blocks this acquired protection, and NPD1, but not 15-HETE, is able to restore protection. Additionally, the neurotrophin PEDF is synthesized during mild oxidative PC, and it both enhances and mimics oxidative PC, resulting in DHA release and concomitant docosanoid synthesis. PEDF is also blocked by inhibition of 15-LOX-1, suggesting that the longer-term signaling during PC protection against mild oxidative damage is possibly mediated through PEDF signaling. This suggests a critical role for DHA and docosanoids in PC protection.

When treated with lethal concentrations of hydrogen peroxide, RPE cells die by apoptosis indicated by nuclear pyknosis. Apoptotic cell death is mediated by the anti-apoptotic Bcl-2 family of proteins. Bcl-2 has been implicated in three different models of photoreceptor degeneration where Bcl-2 overexpression reduces apoptotic PRC death (Crawford et al. 2001). We have previously demonstrated that NPD1 (which is synthesized during DHA supplementation, OS in RPE cells, and ischemia reperfusion) increases expression of the anti-apoptotic Bcl-2 proteins Bcl-xl and Bfl-1(A1), and downregulates pro-apoptotic Bcl-2 associated X protein (BAX), BH3 interacting-domain death agonist (BID), and cyclooxygenase-2 (COX-2) expression in a concentration-dependent manner (Antony et al. 2010; Jablonski et al. 2001). Moreover, it has been documented that neuronal cultures treated with COX inhibitors increase the expression of Bcl-2, and blocking Bcl-2 reduces the pro-survival effect of COX inhibitors on cultured neurons (Jablonski et al. 2001). In this study, we document that both DHA and AA free pool sizes are increased with eicosanoid and docosanoid synthesis in response to mild oxidative PC, and that DHA enhances and mimics PC protection, suggesting that docosanoids are involved in resolution or the protective phase associated with PC. However, it is important to note that while AA and eicosanoids are stimulated and synthesized in response to PC-stimulus, they appeared to not be necessary for the protective actions of PC. This, along with previous reports implicating eicosanoid involvement in PC (Lu et al. 2014; Patel et al. 2013; Gabel et al. 2001), leads us to believe that AA and eicosanoids may be a result of the PC stressor, but is not necessary for the protective actions of PC. This proposes a critical physiological balance of docosanoids and eicosanoids in which docosanoids favor the likelihood of protection and survival, while eicosanoids do not.

PEDF protects photoreceptors from light damage (Liu et al. 2013) and hippocampal neurons from glutamate excitotoxicity (Kim 2007), and supports normal Müller cell development and glutamine synthetase expression after removal of the retinal pigment epithelium (Sanchez et al. 2012). Even though our understanding of the PEDF signaling mechanism in retina is incomplete, PEDF activates several protective signaling pathways in other tissues; specifically, PEDF stimulates intracellular cascades such as PI3K/Akt and ERK 1 and 2 (Chen et al. 2006), leading to nuclear factor (NF)-κB activation, nuclear translocation, and phosphorylation of p38 (Subramanian et al. 2013; Tombran-Tink and Barnstable 2003). In this study, pretreating RPE cells with PEDF or PEDF fragments for 6 h mimics PC protection. We also show that when DHA is supplemented with PEDF prior to PC, this effect is bolstered. In addition, we show that PEDF and its fragments facilitate docosanoid synthesis, but that only PEDF is able to synthesize NPD1 under these conditions. We also document that PEDF expression increases during PC (Chen et al. 2006) and that the protective actions of PEDF are blocked by 15-LOX-1 inhibition. We have previously demonstrated that when PEDF is administered to RPE cells, NPD1 is synthesized, and we have shown that NPD1 is associated with neuroprotective PI3K/AKT signaling (Faghiri and Bazan 2010). Therefore, since PEDF is expressed in response to mild OS (resulting in 17-HDHA and NPD1 synthesis, which also enhance and mimic PC), this suggests that the protective actions of PEDF may be mediated by docosanoids.

Using a model of brief IPC against lethal bright light (Casson et al. 2003), we corroborate that 5 min of intraocular ischemia can protect photoreceptors from bright light-induced degeneration. We also report that this protective effect stimulates 15-HETE, 17-HDHA, and NPD1, all of which are synthesized within 40 min following the IPC-stimulus. However, interestingly, when photoreceptors are exposed to lethal bright light, these aforementioned mediators are stimulated to a greater extent than if the animals are exposed to IPC prior to lethal bright light. This observation, in conjunction with the effects of network disinhibition by bicuculline/4-AP treatment on prostaglandin synthesis, showed protective actions of bicuculline/AP4 treatment that resulted in a lowering of lipid products 24 h after the initial pharmacologic stimulus (Stark and Bazan 2011). Thus, we hypothesize that the initial stimulus (in this case 5 min of ischemia) upregulates more permanent protection or initiates negative feedback mechanisms that either reduce the amount of lipids released or reduce the degree to which the negative effects of the stimulus affects the cell.

PKC has been shown to be critical for IPC through stimulation of phospholipase C (PLC) (Bugge and Ytrehus 1996a, b; Hu and Nattel 1995) in response to the activation of membrane-bound receptors and the release of inositol triphosphate (IP3) and diacyglycerol (DAG) (Dreixler et al. 2008). However, inhibition of various G-coupled receptors does not block PC, suggesting an alternative pathway for PKC activation (Nishizuka 1995). In vitro studies in cardiac cells have reported that AA released by PLA_2_ activates PKC, which can further activate PLA_2_, and generate a positive feedback system (Henke et al. 1984). It has also been shown that potassium channels (K_ATP_-channels) are responsible for PKC activation during IPC (Müller et al. 1992) and can be activated by 17-HDHA or AA, as well as by HETEs and hydroperoxyeicosatetraenoic acids (HPETEs) (Li et al. 2011). Therefore, it is possible that DHA and docosanoids (PUFAs) stimulate K_ATP_ channels in PKC-induced PC since the release of IP3-K/Akt signaling has been associated with DHA and NPD1-mediated protection (Zhao et al. 2011). For this reason, DHA and/or docosanoids (specifically NPD1) may stimulate K_ATP_ channels during PKC-mediated PC, further suggesting that PC protection is mediated, at least in part, through docosanoid synthesis and action.

Lethal bright light results in a buildup of cytotoxic oxidative products, overwhelming photoreceptor protective mechanisms and homeostasis, resulting in cell death (Bazan 2006b). However, when animals are exposed to moderate light or reared in bright light, photoreceptors become resistant to subsequent lethal light exposure (Zhao et al. 2013; Chollangi et al. 2009; Roth et al. 1998). This protective response has been shown to be mediated by trophic factor actions, mainly leukemia-inducing factor (LIF) (Li et al. 2001). We have previously demonstrated that DHA-derived NPD1 is synthesized during trophic factor supplementation and promotes RPE cell survival (Mukherjee et al. 2007), while other research groups have shown that DHA-derived 17-HDHA is associated with PEDF expression (Astarita et al. 2010; Cortina et al. 2010). In this study, we corroborate that moderate to light PC protects photoreceptors from subsequent lethal bright light exposure, but we also demonstrate that while this phenomenon results in 17-HDHA and NPD1 synthesis, it does not result in 15-HETE synthesis. Our in vitro data demonstrate that 17-HDHA and NPD1 enhancement and mimicking take place during the oxidative PC response in RPE cells, while Subramanian et al. 2013 showed that PEDF protects photoreceptors from light-induced PRC death. Therefore, it is reasonable to suggest that these signaling mediators are synthesized in the retina to mitigate daily light-induced or metabolic OS.

In our studies, oxidative, ischemic, and light PC protected RPE and photoreceptors from lethal stressors when compared with non-preconditioned controls. One limitation to our study is that we examined ARPE-19 cells, which, while human in nature, are a spontaneously immortalized cell line that may behave differently than human cells in vivo. However, it is important to mention that we show that primary RPE cells also are protected from PC and that DHA enhances this protection (Fig. 2d). Another limitation to our study is that we only used male animals, when females may have had an enhanced or stunted response to PC. However, to our knowledge most in vivo studies of PC in retina use male animals, and the use of females would confound any interpretations relevant to the current literature.

## Conclusions

Our findings demonstrate that the omega-3 PUFA DHA and the induction of docosanoid synthesis is necessary for PC protection, and thus daily survival, of photoreceptor and RPE cells, both in vivo and in vitro. Our results also show that the protective actions of PEDF involve synthesis and action of 15-LOX products. Our findings support the proposed concept that DHA and docosanoids are responsible for activating sustained cellular mechanisms that elicit long-term PC protection. Since omega-3 PUFA impairments are associated with neuroinflammation, which contributes to neuronal cell dysfunction and death, enhancing the synthesis of docosanoids may provide an opportunity for halting or ameliorating debilitating retinal degenerative diseases.

## Electronic supplementary material

Below is the link to the electronic supplementary material.

Supplementary material 1 (PDF 246 kb)

Supplementary material 2 (PDF 355 kb)

Supplementary material 3 (PDF 513 kb)

Supplementary material 4 (PDF 5549 kb)

Supplementary material 5 (DOCX 15 kb)
