# Supplementary figures and images for: Retinal Pigment Epithelium and Photoreceptor Preconditioning Protection Requires Docosanoid Signaling
Source: Cell Mol Neurobiol. 2017 Nov 24;38(4):901–17. doi: 10.1007/s10571-017-0565-2 (PMC5882642; doi:10.1007/s10571-017-0565-2)

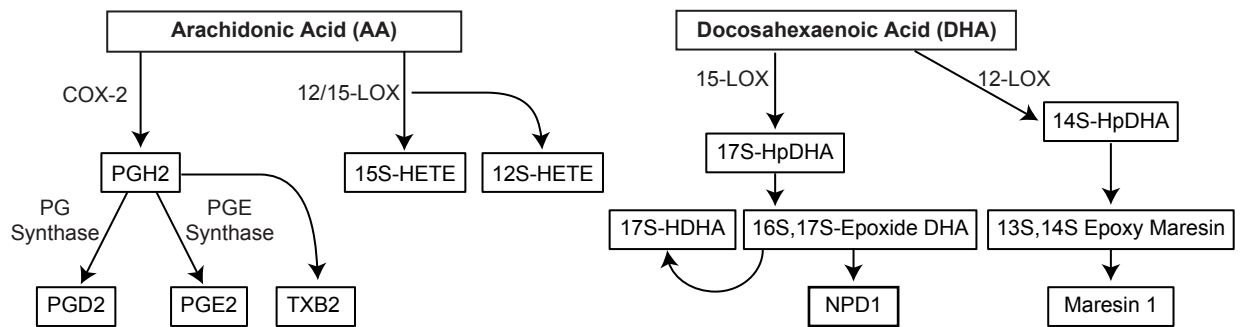

Supplement: Supplementary file 1 — Supplementary material 1 (PDF 246 kb) [file 10571_2017_565_MOESM1_ESM.pdf]

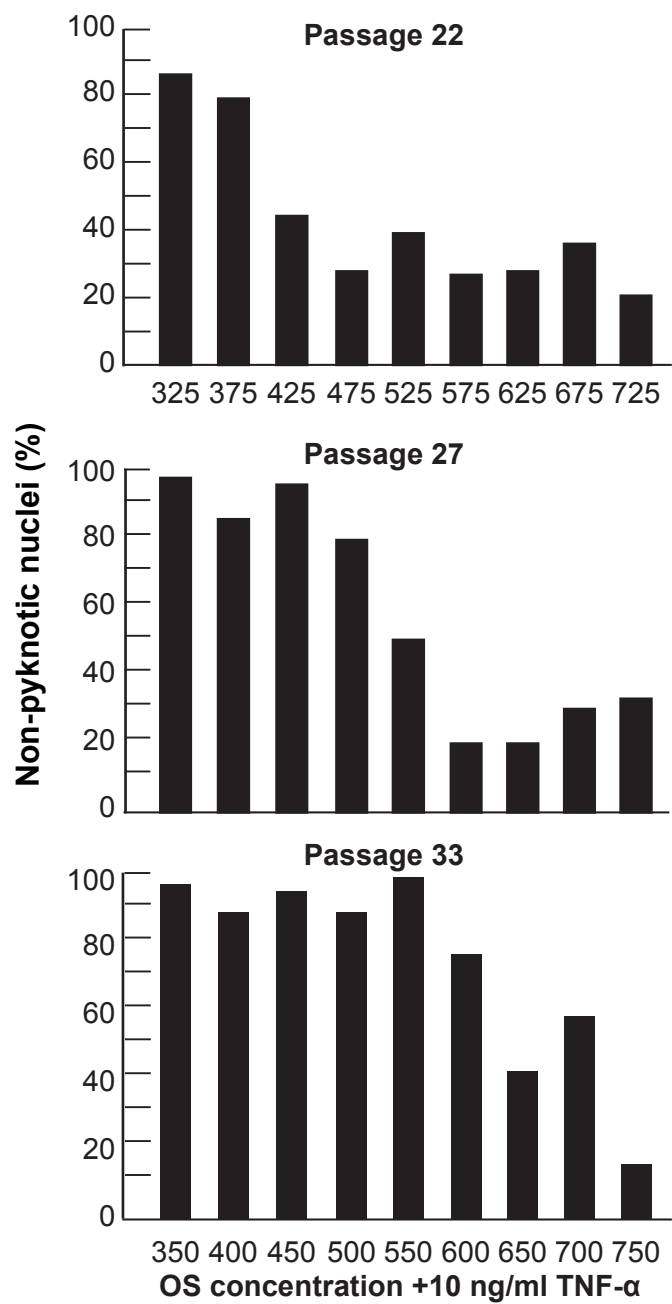

Supplement: Supplementary file 2 — Supplementary material 2 (PDF 355 kb) [file 10571_2017_565_MOESM2_ESM.pdf]

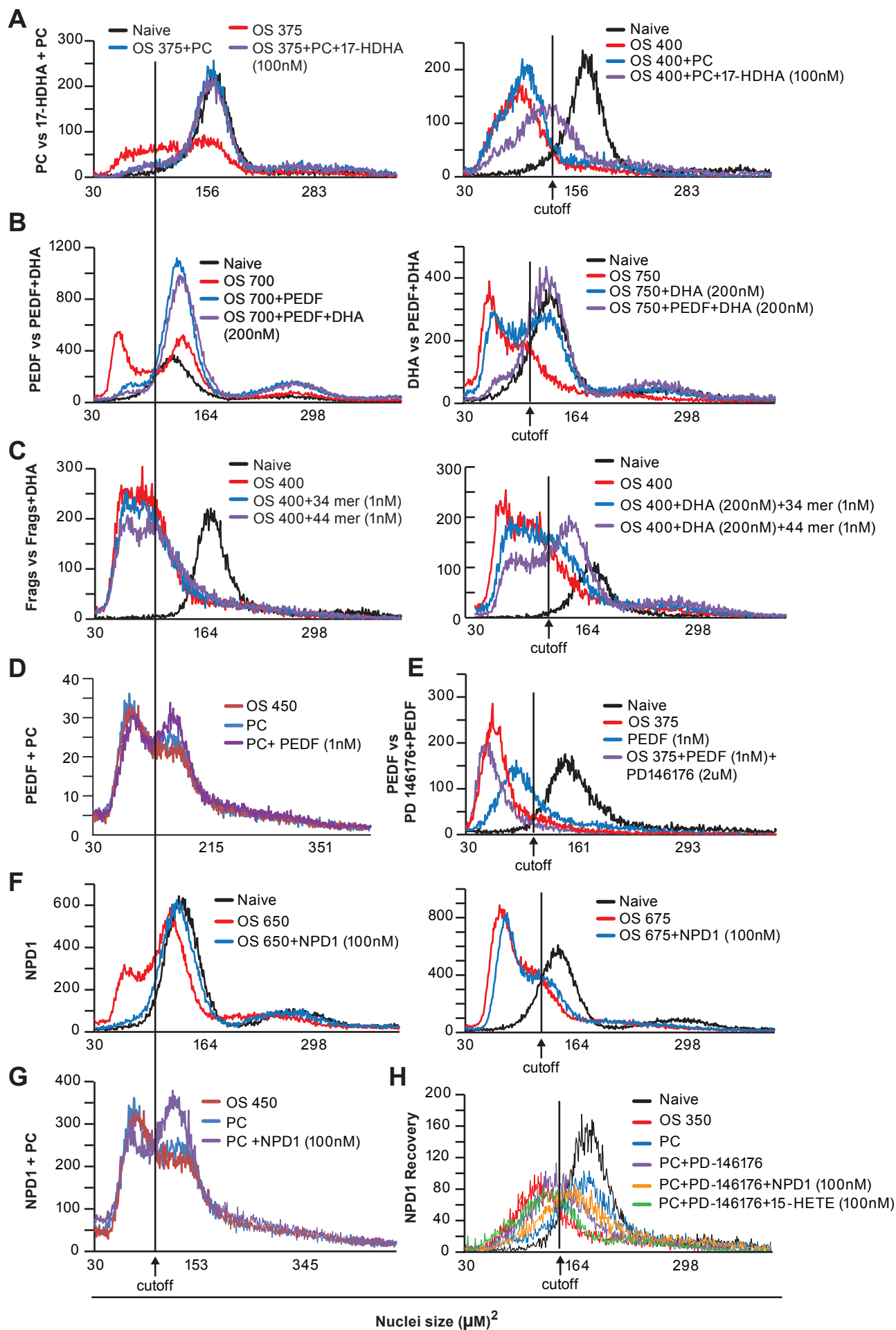

Supplement: Supplementary file 3 — Supplementary material 3 (PDF 513 kb) [file 10571_2017_565_MOESM3_ESM.pdf]
