# Supplementary material for: Retinal Pigment Epithelium and Photoreceptor Preconditioning Protection Requires Docosanoid Signaling
Source: Cell Mol Neurobiol. 2017 Nov 24;38(4):901–17. doi: 10.1007/s10571-017-0565-2 (PMC5882642; doi:10.1007/s10571-017-0565-2)
Supplement: Supplementary file 4 — Supplementary material 4 (PDF 5549 kb) [file 10571_2017_565_MOESM4_ESM.pdf]

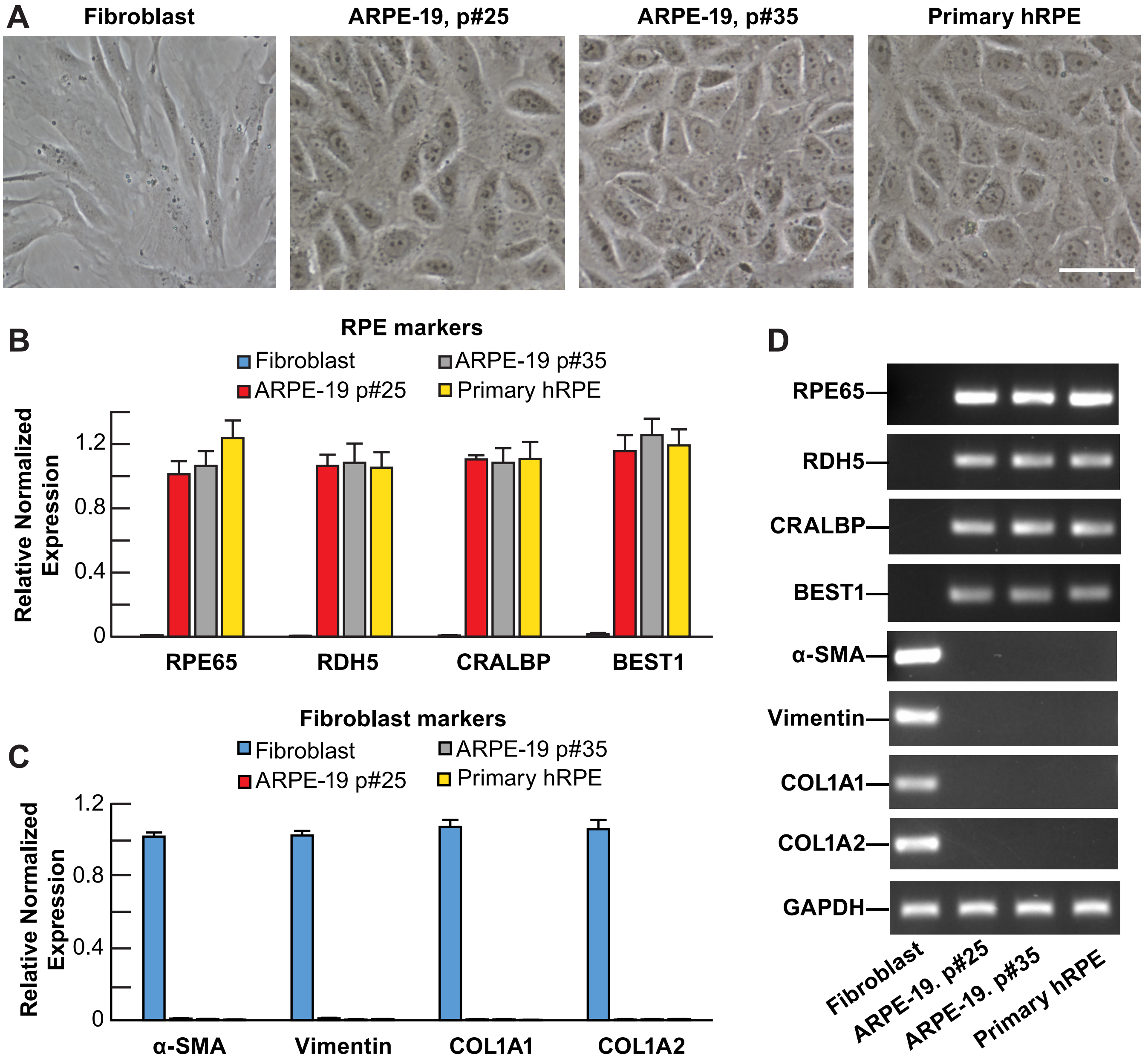


**E**

| Human Gene | Abbreviation | Primer | Sequence |
|---|---|---|---|
| Retinoid Isomerohydrolase | RPE65 | F | CAATGGGTTTCTGATTGTGGA |
| | | R | CCAGTTCTCACGTAAATTGGCTA |
| Retinol Dehydrogenase 5 | RDH5 | F | TGGGTGGAGATGCACGTTAAG |
| | | R | GTGTGGGTCCGATGATACCAG |
| Cellular Retinaldehyde-Binding Protein | CRALBP | F | CAAAGCCATCCACTTCATCCACCA |
| | | R | AAGTCAGAGGGCAGGATGTTCTCA |
| Bestrophin 1 | BEST1 | F | CCTTTATGGGCTCCACCTTCAACATC |
| | | R | CAGTAGTTTGGTCCTTGAGTTTGCCC |
| Alpha smooth muscle actin | α-SMA | F | GTGTTGCCCCTGAAGAGCAT |
| | | R | GCTGGGACATTGAAAGTCTCA |
| Vimentin | VIM | F | TCTCTGAGGCTGCCAACCG |
| | | R | CGAAGGTGACGAGCCATTTCC |
| Collagen Type I Alpha 1 Chain | COL1A1 | F | CGGTGTGACTCGTGCAGC |
| | | R | ACAGCCGCTTCACCTACAGC |
| Collagen Type I Alpha 2 Chain | COL1A2 | F | GTTGCTGCTTGCAGTAACCTT |
| | | R | AGGGCCAAGTCCAACTCCTT |
| Glyceraldehyde-3-Phosphate Dehydrogenase | GAPDH | F | TGGACCTGACCTGCCGTCTA |
| | | R | CCCTGTTGCTGTAGCCAAATTC |
