# Supplementary material for: Retinal Pigment Epithelium and Photoreceptor Preconditioning Protection Requires Docosanoid Signaling
Source: Cell Mol Neurobiol. 2017 Nov 24;38(4):901–17. doi: 10.1007/s10571-017-0565-2 (PMC5882642; doi:10.1007/s10571-017-0565-2)
Supplement: Supplementary file 5 — Supplementary material 5 (DOCX 15 kb) [file 10571_2017_565_MOESM5_ESM.docx]

**Retinal pigment epithelium and photoreceptor preconditioning protection requires docosanoid signaling**

**Running title: Retina preconditioning is mediated by docosanoid signaling in males**

Eric J. Knott^1^, William C. Gordon^2^, Bokkyoo Jun^2^, Khanh Do^2^, Nicolas G. Bazan^2^

^1^Pennington Biomedical Research Center, Louisiana State University, Baton Rouge, Louisiana, 70808, USA

^2^Neuroscience Center of Excellence, School of Medicine, Louisiana State University Health New Orleans, New Orleans, Louisiana, 70112, USA

Correspondence should be addressed to Dr. Nicolas G. Bazan, Neuroscience Center of Excellence, School of Medicine, Louisiana State University Health New Orleans, New Orleans, Louisiana, 70112, USA. E-mail: [nbazan@lsuhsc.edu](mailto:nbazan@lsuhsc.edu)

**Supplementary Figure descriptions.**

**Figure S1**

**Arachidonic acid and Docosahexaenoic acid mediator pathways.**

Summary of the stress-induced pathways of omega-6 arachidonic acid (AA) and omega-3 docosahexaenoic acid (DHA). Molecular products are indicated in boxes and relevant enzymes are indicated by the arrows.

**Figure S2**

Representative lethal OS stress concentration curves for non-pyknotic nuclei of ARP-19 cells of passage 22, 27, and 33.

**Figure S3**

**Nuclei size Frequency Plots**

Nuclear size frequency histogram plots for RPE cells treated with (A) 17 -HDHA ± PC (B) DHA and PEDF (C) PEDF Fragments 34 or 44-mer ± DHA (D) PEDF ± PC (E) PEDF ± PD 146176 (F) NPD1 (G) NPD1 ± PC (H) MPC + PD 146176 + 15 HETE or NPD1. All cells were challenged with OS for 16 hours prior to unbiased cell survival quantification.

**Figure S4**

**ARPE-19 and primary-human Retinal Pigment Epithelial cells (hRPE) express hRPE cell gene characteristics.**

Total RNA was isolated from human fibroblasts (Lifeline Cell Technology, Frederick, MD; #FC-0024), APRE-19 cells, and human Primary RPE cells using RNeasy Plus Mini Kit (Qiagen). 1000 ng of RNA was reverse transcribed into cDNA with iScript cDNA synthesis Kit (Bio-Rad). Expression levels were measured using quantitative BrightGreen 2X qPCR MasterMix (Applied Biological Materials Inc., Richmond, British Columbia, Canada) in a CFX-384 PCR system (Bio-Rad). (A) Example micrographs of fibroblasts, APRE-19 cells (passages 25 and 33), and primary hRPE cells (ABC cells, passage 4). (B) Real-time PCR quantification of primary human RPE and (C) human fibroblast gene expression which was (D) loaded on 2% agarose gels. (E) Primer sequences. Data was normalized using Delta-Delta-Ct (∆∆Ct). All Results are expressed as means ± SEM, n=3, Scale Bar= 50 μm.
